# Functional Polymorphisms in FAS/FASL System Increase the Risk of Neuroblastoma in Chinese Population

**DOI:** 10.1371/journal.pone.0071656

**Published:** 2013-08-12

**Authors:** Wei Han, Yuling Zhou, Rong Zhong, Chen Wu, Ranran Song, Li Liu, Li Zou, Yan Qiao, Kan Zhai, Jiang Chang, Liming Huang, Li Liu, Xuzai Lu, Jiao Lou, Dianke Yu, Wen Tan, Jinzhe Zhang, Huanmin Wang, Xiaoping Miao

**Affiliations:** 1 Department of Surgical Oncology, Beijing Children's Hospital affiliated to Capital Medical University, Beijing, China; 2 Department of Etiology and Carcinogenesis, Cancer Institute, Chinese Academy of Medical Sciences and Peking Union Medical College, Beijing, China; 3 Department of Epidemiology and Biostatistics and MOE Key Lab of Environment and Health, School of Public Health, Tongji Medical College, Huazhong University of Science and Technology, Wuhan, China; 4 Department of Maternal and Child Health, School of Public Health, Tongji Medical College, Huazhong University of Science and Technology, Wuhan, China; 5 Department of Epidemiology and Biostatistics and Guangdong Key Lab of Molecular Epidemiology, School of Public Health, Guangdong Pharmaceutical University, Guangzhou, China; Johns Hopkins University, United States of America

## Abstract

The FAS and FASL system plays a substantial role in apoptosis and immune escape of cells. Three polymorphisms located in the promoter regions of *FAS* (-1377G/A and -670A/G) and *FASL* (-844T/C) have been shown to alter the transcriptional activity of the genes, respectively. This study was conducted to evaluate the effects of these polymorphisms on the susceptibility of neuroblastoma in the Chinese population. A total of 203 patients with neuroblastoma and 411 controls were recruited in this case-control study. Polymerase chain reaction-based restriction fragment length polymorphism (PCR-RFLP) was applied for genotyping. Unconditional logistic regression was used to estimate cancer risk by calculating odds ratios (ORs) and their 95% confidence intervals (95% CIs). It was observed that significantly increased risks of neuroblastoma associated with *FAS* -1377G/A and *FASL* -844T/C polymorphisms, with ORs equal to 1.55 (95% CI, 1.10–2.20) for *FAS* -1377 A allele and 2.90 (95% CI, 2.04–4.12) for *FASL* -844CC genotype carriers compared with non-carriers, respectively. However, no association was found between the polymorphisms of *FAS* -670A/G and risk of neuroblastoma. In addition, the cumulative effect of *FAS* and *FASL* polymorphisms on risk of neuroblastoma was observed (*P* for trend = 2.502×10^−10^), with OR for the carriers of both *FAS* -1377A allele and *FASL* -844CC genotypes equaled to 3.95 (95% CI, 2.40–6.51). This work reveals that polymorphisms of *FAS* -1377G/A and *FASL* -844T/C but not *FAS* -670A/G are associated with risk of neuroblastoma in Chinese. These findings support the hypothesis that genetic polymorphism in FAS/FASL death system may influence individual susceptibility to neuroblastoma.

## Introduction

Neuroblastoma (NB) is a solid tumor derived from primitive sympathetic nervous system that occurs in around 1 in 7000 live births worldwide and accounts for 7–10% of all childhood cancers. About half of all NB arise in the adrenal medulla and the rest originate in paraspinal sympathetic ganglia, thus typically presenting as mass lesions in the neck, chest, abdomen or pelvis, although the clinical presentation is highly variable. With diverse and dramatic clinical behaviors, though a substantial proportion of affected individuals may have spontaneous regression and favorable clinical outcomes even with no or minimal therapy, most of the older patients have extensive or metastatic disease at the time of diagnosis. Overall prognosis of the latter children has been poor despite of intensive therapy [1]–[3].

Accumulative evidence shows the involvement of genetic factors predisposing to NB. Approximately 1% of NB patients present with a family history and in consistence with other hereditary cancer syndromes [4]. Moreover, the concordance for NB in some twins during infancy emphasizes the genetic roles in the development of NB [3]. Subsequently, Mossé et al [5] recently reported that activating mutations in the anaplastic lymphoma kinase oncogene account for most cases of familiar NB. In terms of sporadic NB, alike adult cancer [6]–[12], common genetic variants, which individually have a modest effect in susceptibility, may also play substantial role in the risk of NB. In the last decade, candidate gene approaches have been made to identify several genetic risk factors for NB [13]–[15]. Intriguingly, the recent genome wide association studies (GWAS) further identify single nucleotide polymorphisms (SNP) within the genes FLJ22536 and BARD1 are associated with the increased risk of NB [2], [16]. However, the genetic basis underlying NB remains uncompleted dissected.

Apoptosis plays an important role in multiple physiological processes, such as modifying the developing organism and eliminating unwanted cells or potentially dangerous cells during the entire process of individual development. One of the most important advances in basic cancer research demonstrates that the acquired ability to resist apoptosis is a common hallmark of almost all types of malignant diseases and mutation in the components of apoptosis pathways is one of the pivotal mechanisms in the development of cancer [17]. As the initiator of death pathway, FAS, which is a cell surface receptor and plays a crucial role in apoptotic signaling in many cell types, interacts with its natural ligand (FASL), a member of tumor necrosis factor superfamily, to initiate the death signal cascade, which results in apoptotic cell death [18]. There is compelling evidence demonstrating that reduced expression of FAS and/or increased expression of FASL have been detected in many types of human cancer including NB, indicating the aberrant expression of FAS/FASL system might act as a mechanism for tumor cells to escape from the host immune system [19], [20].

Moreover, there is growing evidence indicating that potentially functional polymorphisms of FAS/FASL system could act as low susceptibility factors and modify the phenotype of cancer [21]–[29]. The -1377G to A and -670A to G transitions in the promoter region of *FAS* disrupt an Sp1 and a STAT1 transcription factor binding site, respectively, which diminish the promoter activity and consequently down-regulate the gene expression [30], [31]. Regarding *FASL*, a T to C transition at position -844 in the promoter region has been reported to be located in a binding motif for another transcription factor, CAAT/enhancer-binding protein β. It has been shown that the -844C allele is associated with significantly enhanced basal expression of FASL compared with the T allele [32]. Interestingly, the authors’ previous studies found the combining effects of *FAS* and *FASL* polymorphisms on risk of esophageal squamous cell carcinoma and lung cancer [26], [28].

In view of the role played by FAS/FASL system in the development of NB, and the presence of the risk alleles of these two genes associated with the susceptibility of adult cancer, we hypothesize that *FAS/FASL* polymorphisms are likely to have a joint effect in conferring susceptibility to NB in Chinese population.

## Materials and Methods

### Study Subjects

To investigate the association between SNPs of *FAS* or *FASL* and NB, 203 patients with NB and 411 controls were enrolled in this case-control study. All patients were recruited from the Beijing Children’s Hospital (Beijing, China) without gender or age restriction. All controls were adults (age from 36 to 65 years) and randomly selected from physical examination database conducted in the Beijing and nearby area during the same time as patients were recruited. The selection criteria for the controls included no history of cancer and matching to cases on gender. In this study, 411 controls were also enrolled to match the cases. At recruitment, written informed consent was obtained from each subject or their parents and legal guardians. And personal data from each participant regarding demographic characteristics such as gender and age were collected by questionnaire. This study was approved by the institutional review board of the Beijing Children’s Hospital and Chinese Academy of Medical Sciences Cancer Institute.

### Polymorphism Analysis

Genomic DNA was extracted from 2 ml peripheral blood devoted by each participant in the study and stored at −30°C refrigerator until use. According to relative researches reported previously [26]–[28], polymerase chain reaction-based restriction fragment length polymorphism (PCR-RFLP) method was performed to genotype these three SNPs, *FAS* (-1377G/A and -670 A/G) and *FASL* (-844T/C), in the promoter region of death pathway genes to determine the association between genetic variations and susceptibility to NB.

### Statistical Analysis

Difference in gender between case patients and control population was examined by two-side χ^2^ test. The associations between the polymorphisms and risk of NB were estimated by odds rations (ORs) and their 95% confidence intervals (95% CIs) calculated by using unconditional multivariate logistic regression analysis. Because the minor allele frequencies (MAF) for *FAS* -1377G/A, -670A/G and *FASL* -844T/C were 0.33, 0.36 and 0.35 in controls, we calculated the power for the sample size of 203 patients and 411 controls as follows: for SNPs with MAF of 0.33, the power for our sample size to detect an OR of 1.50 is 0.64. In addition, cumulative effect of *FAS* and *FASL* polymorphisms on risk to develop NB was also investigated. The OR was adjusted by gender where it was appropriated and a P value less than 0.05 was considered significant. All analyses were two-sided and performed using Statistical Analysis System (Version 6.12, SAS Institute, Cary, NC).

## Results

### Patient Characteristics

Of the 203 patients with NB and 411 controls in the study, the distribution of gender among subjects was summarized in Table 1. There was no significant difference between case and control participants in term of gender examined by two-side χ^2^ test. Male subjects accounted for 64.0% of the total cases compared with 68.1% of all the controls. About 66.5% of the patients were at their 12 to 60 months of age. 30 patients (14.8%) were less than 12 months and 35 patients (17.2%) were older than 60 months. In view of clinical stages, there were 28 (13.8%), 42 (20.7%), 57 (28.1%), 59 (29.1%) and 12 (5.9%) patients fell in stage I, II, III, IV and 4s, respectively. Staging of disease and diagnosis followed INSS criteria [33]. The clinical stage of the other 4 patients was not clear. The primary site of tumor of 181 patients was abdominal region, including 86 (42.4%) in adrenal gland, 83 (40.9%) in retroperitoneal region, 10 (4.9%) in pelvic cavity and 2 (1.0%) in sacrococcygeal region. The sites of origin of another 4 patients were the neck region and of the other 18 patients were not clear.

**Table 1:** Distributions of select characteristics by case-control status.

Variable	Cases (*n* = 203)	N(%)	Controls (*n* = 411)	N(%)		*P* a
Gender						0.312
Male	130	(64.0)	280		(68.1)	
Female	73	(36.0)	131		(31.9)	
Age						
<12 months	30	(14.8)				
12–60 months	135	(66.5)				
>60 months	35	(17.2)				
unknown	3	(1.5)				
Clinical Stage						
I	28	(13.8)				
II	42	(20.7)				
III	57	(28.1)				
IV	59	(29.1)				
4 s	12	(5.9)				
unknown	5	(2.5)				
Site of origin						
neck	4	(2.0)				
abdomen						
adrenal gland	86	(42.4)				
retroperitoneal region	83	(40.9)				
pelvic cavity	10	(4.9)				
sacrococcygeal region	2	(1.0)				
unknown	18	(8.9)				

a Two-sided χ^2^ test.

### Association between SNPs and Risk of NB

Genotype and allele frequencies of *FAS* and *FASL* among cases and controls, and their contribution to risk of NB are showed in Table 2. The allele frequencies for *FAS* -1377A, -670G and *FASL* -844C were 0.33, 0.36 and 0.65 in controls, compared with 0.39, 0.41 and 0.80 in cases, respectively. Genotype frequencies of *FAS* -1377G/A and -670A/G were distributed analogously between cases and controls and the difference of distribution was not significant in this study. However, it was observed totally different distribution of *FASL* -844T/C genotypes among patients with NB and controls. Two hundred and twelve [212 (51.6%)] controls carried *FASL* -844TC genotype, compared with 51(28.1%) NB patients carrying FASL -844TC genotype. Meanwhile, the CC genotype was more prevalent among all the case patients than the controls. Of all patients, 133 (65.5%) cases carried CC genotype, whereas, only 162 (39.4%) controls were CC genotype carriers.

**Table 2:** Genotype and allele frequencies of *FAS* and *FASL* among cases and controls, and their contributions to risk of neuroblastoma.

Genotype	Controls (*n* = 411) N(%)		Cases (*n* = 203) N(%)		OR^a^ (95% CI)
*FAS* -1377G/A					
*GG*	186	(45.3)	71	(35.0)	1.00
*GA*	180	(43.8)	105	(51.7)	1.55 (1.07–2.23)
*AA*	45	(10.9)	27	(13.3)	1.58 (0.91–2.74)
GG	186	(45.3)	71	(35.0)	1.00
*GA*+*AA*	225	(54.7)	132	(65.0)	1.55 (1.10–2.20)
*A* allele frequency	0.33		0.39		
*FAS* -670A/G					
*AA*	163	(39.7)	67	(33.0)	1.00
*AG*	197	(47.9)	104	(51.2)	1.30 (0.90–1.88)
*GG*	51	(12.4)	32	(15.8)	1.53 (0.90–2.59)
*AA*	163	(39.7)	67	(33.0)	1.00
*AG*+*GG*	248	(60.3)	136	(67.0)	1.35 (0.94–1.92)
*G* allele frequency	0.36		0.41		
*FASL* -844T/C					
*TT*	37	(9.0)	13	(6.4)	1.00
*TC*	212	(51.6)	57	(28.1)	0.76 (0.38–1.52)
*CC*	162	(39.4)	133	(65.5)	2.31 (1.18–4.52)
*TT*+*TC*	249	(60.6)	70	(34.5)	1.00
*CC*	162	(39.4)	133	(65.5)	2.90 (2.04–4.12)
*C* allele frequency	0.65		0.80		

a ORs and 95% CIs were calculated by unconditional logistic regression adjusting for gender where it was appropriate.

Unconditional logistic regression model was used to estimate the association between the genotype and risk of NB (Table 2). Individuals carrying the *FAS* -1377 GA or AA genotype had an OR of 1.55 (95% CI = 1.10–2.20) compared with individuals with the GG genotype. For the *FASL* -844T/C SNP, *FASL* -844CC genotype presented 2.31-fold elevated risk (95% CI = 1.18–4.52) for NB compared with TT genotype although the heterozygous genotype was not associated with the risk. In addition, the *FASL* -844CC genotype carrier was 2.90-fold (95% CI = 2.04–4.12) risk to develop NB compared with TT or TC genotype carrier.

Table 3 showed the cumulative effect of the *FAS -*1377G/A and *FASL* -844T/C on the risk of NB. Since the two polymorphisms in *FAS*, -670A/G and -1377G/A, were linkaged tightly, only *FAS -*1377G/A site was selected for analysis. Compared to individuals who carried *FASL* -844T allele and *FAS -*1377 GG genotype, subjects carrying *FASL* -844CC and *FAS -*1377 GG genotype were found that their OR for NB was 2.45 (95% CI = 1.40–4.28). However, individuals carrying the combination of *FAS* -1377A and *FASL* -844T allele did not present significantly increased risk of NB. Moreover, for the subjects who carried the combination of *FAS* -1377A allele and *FASL* -844CC, the OR increased to 3.95 (95% CI = 2.40–6.51). These results demonstrated that the cumulative effect of *FAS -*1377G/A and *FASL* -844T/C polymorphisms might increase the risk of NB in this study (*P* for trend = 2.502×10^−10^).

**Table 3:** Risk of neuroblastoma associated with the *FAS -*1377G/A genotypes by *FASL* -844T/C genotypes.

Genotype^b^		Patients (*n* = 203)		Controls (*n* = 411)		OR^a^ (95% CI)
*FASL* -844T/C	*FAS* -1377G/A	*n*	(%)	*n*	(%)	
*TT*+*TC*	*GG*	29	(14.3)	117	(28.5)	1.00
*TT*+*TC*	*GA+AA*	41	(20.2)	132	(32.1)	1.27 (0.74–2.17)
*CC*	*GG*	42	(20.7)	69	(16.8)	2.45 (1.40–4.28)
*CC*	*GA+AA*	91	(44.8)	93	(22.6)	3.95 (2.40–6.51)

a ORs and 95% CIs were calculated by unconditional logistic regression adjusting for gender.

b Test for linear trend, *P* = 2.502×10^−10^.

## Discussion

This molecular genetics study examined the association between genetic polymorphisms in apoptotic pathway FAS/FASL system, alone or in combination, and risk of the NB in Chinese population. On the basis of analysis of 203 case patients and 411 gender-matched normal controls, in accordance with the previous studies on esophageal squamous cell carcinoma and lung cancer [26], [28], it was demonstrated that subjects carrying the *FAS* -1377AA or GA genotype presented an increased risk for developing NB compared with the non-carriers. Meanwhile, the subjects of *FASL* -844CC showed a more than two-time higher risk to NB (OR = 2.31, 95% CI = 1.18–4.52) than the individuals carried *FASL* -844TT. Moreover, a cumulative effect of *FAS* -1377G/A and *FASL* -844T/C polymorphisms on risk of neuroblastoma was also observed (table 2). To the best of our knowledge, this was the first study to investigate the polymorphisms in FAS/FASL system and risk of NB.

NB is the most common extracranial solid tumor in childhood and the most common cancer in infancy [1]–[3]. Although the reason for occurrence of NB has not yet been fully clarified, no environment factors are known clearly to the development of NB. Whiles growing body of evidence indicates that an individual’s genetic makeup might determine the susceptibility for NB [5], [14], [15]. Results of this study demonstrating the individual and cumulative effects of the variant genotypes of *FAS* -1377G/A and *FASL* -844T/C in NB susceptibility were biologically plausible. Firstly, FAS/FASL system plays a crucial role in the apoptosis process. FAS interacts with FASL to initiate a catalytic caspase cascade, which transduces the apoptosis signal and promotes apoptosis of cells. In addition, there are a large number of studies that had shown that up-regulation of FASL and/or down-regulation of FAS expression were observed in many human cancers and were associated with development of cancers, including NB [20], [23], [24], [34]–[38]. Secondly, the investigated polymorphisms in the FAS/FASL system had been shown to be of functional significance. The *FAS* -1377G/A and *FASL* -844T/C variations in the promoter region occurred within Sp1 and C/EBPβ transcription factor binding site, and thus decrease FAS expression by diminishing gene promoter activity and increased expression of FASL, respectively [30], [32]. These data provided very plausible molecular mechanisms through which the up-regulation of FASL and down-regulation of FAS, resulting from genetic polymorphism of FAS/FASL system might foster increased susceptibility to NB and also helped the authors to gain an insight into the pathogenesis of NB. Moreover, with the important role of FAS/FASL system in apoptosis pathway in mind, one might expect that individuals who carried both the *FAS* and *FASL* risk genotypes concomitantly may be more susceptible to the disease. It might be expected that the transformed cells which carried the *FASL* -844CC genotype with high level of FASL might create an immunoprivileged site by killing cytotoxic immune cells, thus escaping host immunosurveilance; on the other hand, reduced expression of FAS due to the *FAS* -1377AA genotype might assist the transformed cells to evade FAS-mediated cell death.

This study has limitations, some of which could not be overcome. First of all, the patients might not be representatives of total NB patients and inherent selection bias might not be completely excluded in the study since they were recruited from only one hospital and the controls were selected from the community population. Secondly, there are several genetic features of NB, such as MYCN amplification and ploidy status, which have been identified to be correlated with the clinical outcome that be needed for further clarification in these 203 patients to have the findings more exact. Thirdly, would be the sample size issue. This case-control study might be, to the best of our knowledge, the largest one about NB genetic susceptibility in Chinese population. Nevertheless, the statistical power is still relatively low to detect the modest effect of common genetic variation by 203 patients and 411 controls; further larger study is warranted to verify the results.

The motivation for this study was the paucity of information on NB pathogenesis in Chinese population. Patients were enrolled with NB from the Beijing children’s hospital, the largest center from NB in China, and provided convincing evidences that the functional polymorphisms in the promoter regions of FAS/FASL system were associated with the susceptibility of NB. These results may also support the hypothesis that FAS/FASL-triggered apoptosis pathway plays an important role in NB carcinogenesis. However, with the recent progresses in the genome wide association studies and sequencing to detect the genetic susceptibility of complex diseases[39]–[42], especially NB, this current study warrant to be integrated with more complex genetic markers in future.
